# Supplementary figures and images for: The epidemiology and outcomes of central nervous system infections in Far North Queensland, tropical Australia; 2000-2019
Source: PLoS One. 2022 Mar 21;17(3):e0265410. doi: 10.1371/journal.pone.0265410 (PMC8936475; doi:10.1371/journal.pone.0265410)

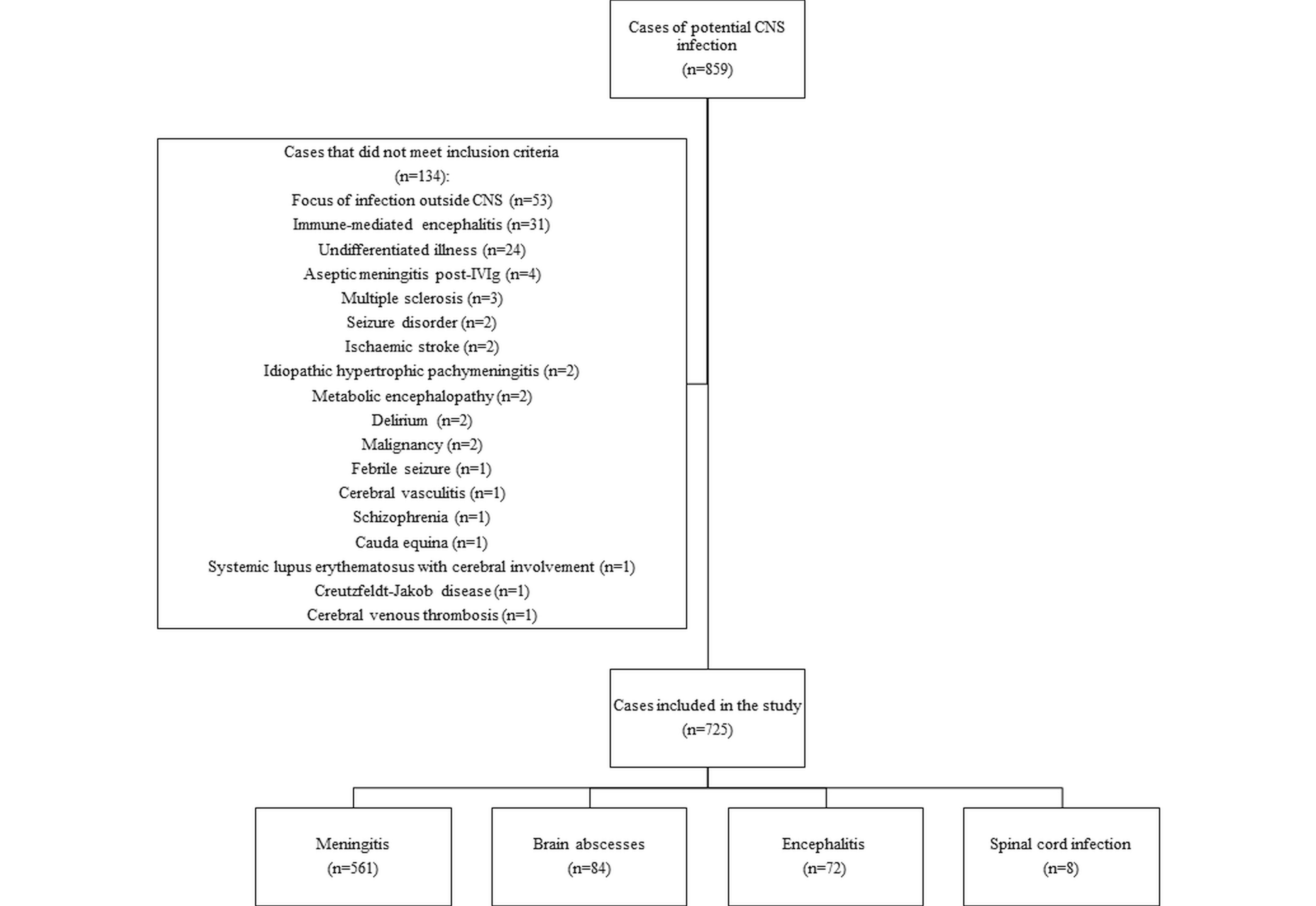

Supplement: S1 Fig — (TIF) [file pone.0265410.s001.tif]

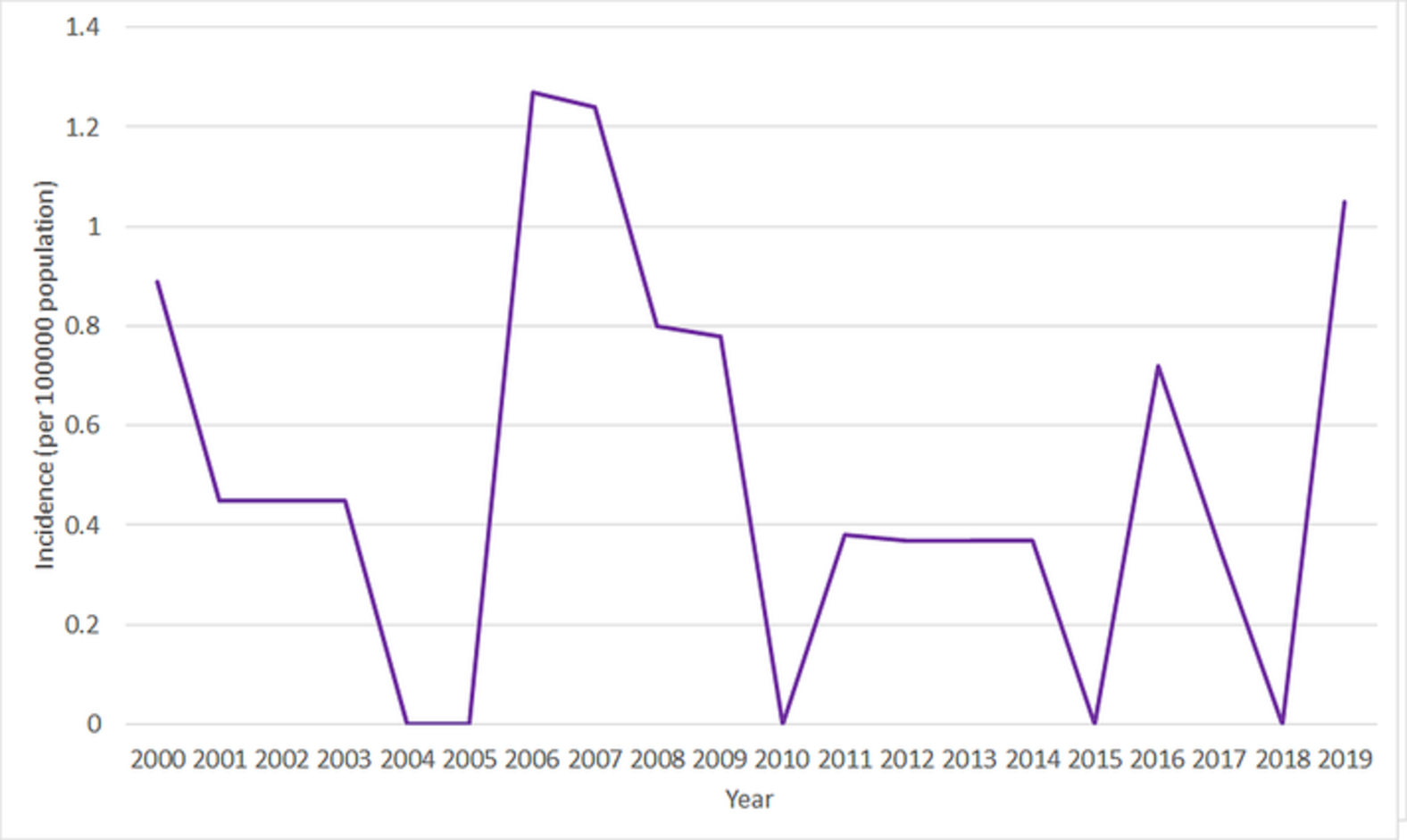

Supplement: S2 Fig — (TIF) [file pone.0265410.s002.tif]

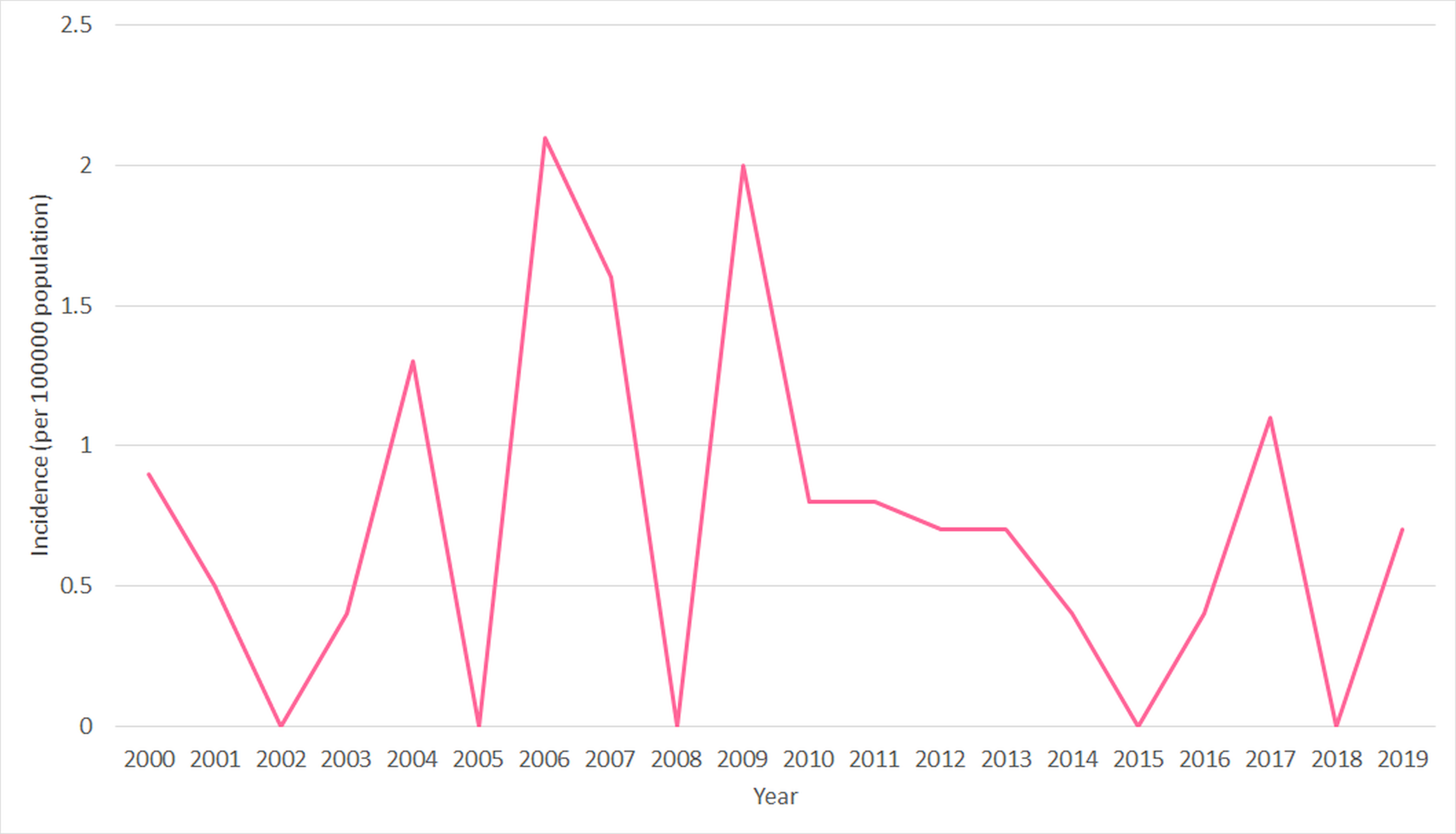

Supplement: S3 Fig — (TIF) [file pone.0265410.s003.tif]
