# Supplementary material for: The epidemiology and outcomes of central nervous system infections in Far North Queensland, tropical Australia; 2000-2019
Source: PLoS One. 2022 Mar 21;17(3):e0265410. doi: 10.1371/journal.pone.0265410 (PMC8936475; doi:10.1371/journal.pone.0265410)
Supplement: S1 Table — (DOCX) [file pone.0265410.s004.docx]

**S1 Table. Incidence per 100,000 local population between 2000 and 2019.**

| **Year** | **All infections** | **Meningitis** | **Encephalitis** | **Abscess** | **Spinal disease** |
| --- | --- | --- | --- | --- | --- |
| 2000 | 12.97 | 1.79 | 8.95 | 0.89 | 1.34 |
| 2001 | 22.97 | 0.90 | 20.72 | 0.90 | 0.45 |
| 2002 | 7.71 | 0.45 | 6.80 | 0.45 | 0.00 |
| 2003 | 9.82 | 0.45 | 8.03 | 1.34 | 0.00 |
| 2004 | 7.91 | 1.76 | 4.83 | 1.32 | 0.00 |
| 2005 | 6.04 | 0.43 | 4.75 | 0.86 | 0.00 |
| 2006 | 11.41 | 1.27 | 9.30 | 0.85 | 0.00 |
| 2007 | 15.64 | 0.82 | 14.00 | 0.82 | 0.00 |
| 2008 | 18.42 | 1.20 | 16.01 | 1.20 | 0.00 |
| 2009 | 11.74 | 0.39 | 9.39 | 1.96 | 0.00 |
| 2010 | 11.94 | 1.93 | 8.48 | 1.54 | 0.00 |
| 2011 | 8.00 | 0.76 | 6.10 | 1.14 | 0.00 |
| 2012 | 16.12 | 1.12 | 13.49 | 1.12 | 0.37 |
| 2013 | 13.29 | 2.21 | 9.23 | 1.85 | 0.00 |
| 2014 | 13.87 | 1.83 | 8.40 | 3.65 | 0.00 |
| 2015 | 13.38 | 1.81 | 7.96 | 3.62 | 0.00 |
| 2016 | 12.88 | 1.07 | 11.45 | 0.36 | 0.00 |
| 2017 | 15.26 | 1.42 | 12.42 | 0.71 | 0.71 |
| 2018 | 14.06 | 2.11 | 10.19 | 1.41 | 0.35 |
| 2019 | 21.60 | 1.74 | 16.73 | 3.14 | 0.00 |
| **p for trend** | 0.09 | 0.22 | 0.06 | 0.12 | 0.84 |
