# Supplementary material for: The epidemiology and outcomes of central nervous system infections in Far North Queensland, tropical Australia; 2000-2019
Source: PLoS One. 2022 Mar 21;17(3):e0265410. doi: 10.1371/journal.pone.0265410 (PMC8936475; doi:10.1371/journal.pone.0265410)
Supplement: S2 Table — (DOCX) [file pone.0265410.s005.docx]

**S2 Table. Pathogens identified in Aboriginal and Torres Strait Islander children and infants.**

| Enterovirus (n=37)  *Neisseria meningitidis* (n=9)  *Haemophilus influenzae* (n=5)  *Streptococcus pneumoniae* (n=5)  *Streptococcus pyogenes* (n=2)  *Staphylococcus aureus* (n=2)  *Salmonella* species (n=2)  Herpes simplex virus-2 (n=1)  *Elizabethkingia (Chryseo.) meningoseptica* (n=1) |
| --- |

A pathogen was not identified in 31/95 (32.6%) cases.
