# Supplementary material for: The epidemiology and outcomes of central nervous system infections in Far North Queensland, tropical Australia; 2000-2019
Source: PLoS One. 2022 Mar 21;17(3):e0265410. doi: 10.1371/journal.pone.0265410 (PMC8936475; doi:10.1371/journal.pone.0265410)
Supplement: S3 Table — (DOCX) [file pone.0265410.s006.docx]

**S3 Table. Pathogens in patients who lived outside Australia.**

|  | **Papua New Guinea (n=32)** | **Other countries outside Papua New Guinea (n=15)** |
| --- | --- | --- |
| **Pathogens** | *Mycobacterium tuberculosis* (n=7)  *Streptococcus pneumoniae* (n=2)  *Haemophilus influenzae* (n=2)  Enterovirus (n=2)  Japanese encephalitis virus (n=1)  Murray Valley encephalitis virus (n=1)  *Burkholderia pseudomallei* (n=1)  *Cryptococcus neoformans* (n=1)  *Cryptococcus gattii* (n=1)  *Klebsiella pneumoniae* (n=1)  *Salmonella Virchow* (n=1)  Epstein Barr virus (n=1)  No pathogen identified (n=11) | *Neisseria meningitidis* (n=2)  Herpes simplex virus-2 (n=2)  Enterovirus (n=2)  *Streptococcus pneumoniae* (n=1)  *Staphylococcus aureus* (n=1)  *Cryptococcus gattii* (n=1)  *Cryptococcus neoformans* (n=1)  *Salmonella Virchow* (n=1)  *Aeromonas hydrophilia* (n=1)  No pathogen identified (n=3) |
