# Supplementary material for: The epidemiology and outcomes of central nervous system infections in Far North Queensland, tropical Australia; 2000-2019
Source: PLoS One. 2022 Mar 21;17(3):e0265410. doi: 10.1371/journal.pone.0265410 (PMC8936475; doi:10.1371/journal.pone.0265410)
Supplement: S4 Table — (DOCX) [file pone.0265410.s007.docx]

**S4 Table. Pathogens identified in immunocompromised patients.**

| *Cryptococcus* (n=6)   - - - *Cryptococcus neoformans* (n=4)     - Non-speciated (n=2)   Varicella zoster virus (n=4)  *Toxoplasma gondii* (n=2)  *Listeria monocytogenes* (n=2)  Enterovirus (n=1)  Herpes simplex virus-1 (n=1)  Herpes simplex virus-2 (n=1)  Human herpesvirus 6 (n=1)  JC virus (n=1)  *Acanthamoeba* species (n=1)  *Mycobacterium tuberculosis* (n=1)  *Nocardia paucivirans* (n=1)  *Kingella kingae* (n=1)  *Propionibacterium acnes* (n=1)  *Salmonella* species (n=1) |
| --- |

A pathogen was not identified in 13/38 (34.2%) immunocompromised patients.
