# Supplementary material for: The epidemiology and outcomes of central nervous system infections in Far North Queensland, tropical Australia; 2000-2019
Source: PLoS One. 2022 Mar 21;17(3):e0265410. doi: 10.1371/journal.pone.0265410 (PMC8936475; doi:10.1371/journal.pone.0265410)
Supplement: S5 Table — (DOCX) [file pone.0265410.s008.docx]

**S5 Table. Serotypes identified in pneumococcal CNS infection.**

| **Year** | **Age** | **Serotype identified** | **Identified serotype present in the vaccines** |
| --- | --- | --- | --- |
| 2001 | 1 | 23F | No |
| 2002 | 44 | 10F | No |
| 2003 | 1 | 6B | No |
| 2006 | 0 | 18C | Yes |
| 2006 | 2 | 19A | Yes |
| 2006 | 47 | 3 | Yes |
| 2007 | 61 | 15C | No |
| 2007 | 10 | 10A | Yes |
| 2008 | 49 | 19F | Yes |
| 2008 | 1 | 18C | Yes |
| 2009 | 75 | 35B | No |
| 2013 | 0 | 18C | Yes |
| 2016 | 46 | 16F | No |
| 2019 | 68 | 3 | Yes |
| 2019 | 48 | 34 | No |
