# Supplementary material for: The epidemiology and outcomes of central nervous system infections in Far North Queensland, tropical Australia; 2000-2019
Source: PLoS One. 2022 Mar 21;17(3):e0265410. doi: 10.1371/journal.pone.0265410 (PMC8936475; doi:10.1371/journal.pone.0265410)
Supplement: S6 Table — (DOCX) [file pone.0265410.s009.docx]

**S6 Table. Pathogens causing CNS infection in patients who died.**

| **Infants (n=5)** | **Children (n=3)** | **Adults (n=24)** |
| --- | --- | --- |
| Methicillin-sensitive *S. aureus* (n=1)  Methicillin-resistant *S. aureus* (n=1)  *Salmonella Virchow* (n=1)  *H. influenzae* (n=1)  *N. meningitidis* (n=1) | *M. tuberculosis* (n=1)  *B. pseudomallei* (n=1)  *C. gattii* (n=1) | *Cryptococcus* species (n=3)  Herpes simplex virus-1 (n=2)  *M. tuberculosis* (n=1)  *B. pseudomallei* (n=1)  *Enterococcus faecalis* (n=1)  *Streptococcus agalactiae* (n=1)  *Streptococcus milleri* (n=1)  JC virus (n=1)  Methicillin-sensitive *S. aureus* (n=1)  No pathogen identified (n=12) |
