# Supplementary material for: The epidemiology and outcomes of central nervous system infections in Far North Queensland, tropical Australia; 2000-2019
Source: PLoS One. 2022 Mar 21;17(3):e0265410. doi: 10.1371/journal.pone.0265410 (PMC8936475; doi:10.1371/journal.pone.0265410)
Supplement: S7 Table — (DOCX) [file pone.0265410.s010.docx]

**S7 Table. Death and disability outcomes stratified by clinical phenotypes, aetiology and demographic characteristics.**

|  | **All n=270** | **No long-term sequelae n=190** | **Died or disability n=80** | **p** |
| --- | --- | --- | --- | --- |
| **Clinical phenotype** | | | | |
| **Meningitis** | 190 (70.4%) | 161 (84.7%) | 29 (36.3%) | <0.0001 |
| **Encephalitis** | 34 (12.6%) | 13 (6.8%) | 21 (26.3%) | <0.0001 |
| **Brain abscess** | 44 (16.3%) | 15 (7.9%) | 29 (36.3%) | <0.0001 |
| **Spinal disease** | 2 (0.7%) | 1 (0.5%) | 1 (1.2%) | 0.51 |
| **Aetiological agent** | | | | |
| **Bacterial** | 75 (27.8%) | 34 (17.9%) | 41 (51.3%) | <0.0001 |
| **Viral** | 112 (41.5%) | 101 (53.2%) | 11 (13.8%) | <0.0001 |
| **Fungal** | 6 (2.2%) | 2 (1.0%) | 4 (5.0%) | 0.07 |
| **Not identified ^a^** | 74 (27.4%) | 52 (27.4%) | 22 (27.5%) | 0.98 |
| **Demographic characteristics** | | | | |
| **Infant** | 88 (32.6%) | 74 (38.9%) | 14 (17.5%) | 0.001 |
| **Child** | 29 (10.7%) | 17 (8.9%) | 12 (15.0%) | 0.14 |
| **Adult** | 153 (56.7%) | 99 (52.1%) | 54 (67.5%) | 0.02 |
| **Indigenous ^b^** | 67/264 (25.4%) | 47/186 (25.3%) | 20/78 (25.6%) | 0.95 |
| **Remote residence ^c^** | 29/257 (11.3%) | 20/183 (10.9%) | 9/74 (12.2%) | 0.83 |

^a^ There were 3 patients with other confirmed diagnoses; 1 patient with *Acanthamoeba* infection who survived with no sequalae and 1 case each of *Toxoplasmosis gondii* infection and gnathostomiasis who both had sequalae.

^b^ Indigenous status was accessible in 264/270 cases.

^c^ Of the 270 cases, 257 occurred in local residents.
