# Supplementary material for: The epidemiology and outcomes of central nervous system infections in Far North Queensland, tropical Australia; 2000-2019
Source: PLoS One. 2022 Mar 21;17(3):e0265410. doi: 10.1371/journal.pone.0265410 (PMC8936475; doi:10.1371/journal.pone.0265410)
Supplement: S8 Table — (DOCX) [file pone.0265410.s011.docx]

**S8 Table. Residual deficits in infants, children and adults after CNS infection, and associated pathogens.**

| **Pathogens** | **Residual deficits** |
| --- | --- |
| Infants | |
| *Salmonella Aberdeen* | Cerebral palsy |
| *Salmonella enteritidis* | Cerebral palsy |
| *Streptococcus agalactiae* | Hearing impairment |
| *Haemophilus influenzae* | Hearing and speech impairment, seizures |
| *Streptococcus pyogenes* | Speech impairment |
| *Citrobacter freundii* | Cerebral palsy, global developmental delay |
| *Streptococcus pneumoniae* | Hearing impairment, seizures, motor impairment |
| Children | |
| *Burkholderia pseudomallei* | Impairment of speech, cognition and motor function, dysphagia, gait instability |
| *Streptococcus pneumoniae* | Hearing impairment |
| *Neisseria meningitidis* | Hearing impairment |
| *Streptococcus pyogenes* | Impairment of cognition, memory and sleep |
| *Leptospira* | Headaches, altered level of consciousness |
| Herpes simplex virus-1 | Movement disorder, seizures, impairment of cognition, behaviour and speech |
| Adults | |
| *Burkholderia pseudomallei* | Impairment of cognition, vision and motor function, facial droop, laryngospasms, bulbar palsy, cerebellar dysfunction |
| *Streptococcus pneumoniae* | Impairment of motor function and hearing, bladder dysfunction |
| *Nocardia paucivirans* | Impairment of speech, motor function and cognition, seizures |
| *Mycobacterium tuberculosis* | Cognitive impairment |
| *Treponema pallidum* | Vision impairment |
| *Streptococcus milleri* | Impairment of cognition, motor and sensory function, gait instability |
| *Listeria monocytogenes* | Hearing and vision impairment |
| *Propionibacterium acnes* | Cognitive impairment, personality change |
| *Staphylococcus epidermidis* | Pain |
| *Escherichia coli* | Headaches |
| *Streptococcus sanguinis, Streptococcus parasanguinis, Microbacterium* sp | Memory impairment |
| *Enterococcus faecalis, Serratia marcescens* | Motor impairment |
| Herpes simplex virus-1 | Impairment of cognition, speech and motor function, seizures, personality change |
| Herpes simplex virus-2 | Sensory impairment, bowel dysfunction |
| Varicella zoster virus | Impaired coordination, facial droop |
| *Cryptococcus gattii* | Impairment of motor function, cognition, vision and speech, gait instability, headaches, |
| *Cryptococcus neoformans* | Impairment of memory, cognition and vision, gait instability, headaches, fatigue |
| *Toxoplasma gondii* | Vision impairment |
| *Gnathostoma* | Impairment of cognition and memory |
