# Supplementary material for: The epidemiology and outcomes of central nervous system infections in Far North Queensland, tropical Australia; 2000-2019
Source: PLoS One. 2022 Mar 21;17(3):e0265410. doi: 10.1371/journal.pone.0265410 (PMC8936475; doi:10.1371/journal.pone.0265410)
Supplement: S9 Table — (DOCX) [file pone.0265410.s012.docx]

**S9 Table. Most common pathogens causing CNS infection and case fatality rates in Far North Queensland, Vietnam and Laos [4, 5].**

|  | **FNQ (2000 – 2019)** | **Vietnam (2007 – 2010)** | **Laos (2003 – 2011)** |
| --- | --- | --- | --- |
| **Most common pathogens** | Enterovirus (30.3%) | *Streptococcus suis* (11.8%) | JEV (8.8%) ^a^ |
|  | *N. meningitidis* (5.0%) | JEV (11.4%) ^a^ | *Cryptococcus species* (6.6%) |
|  | *Cryptococcus species* (4.7%) | *M. tuberculosis* (9.8%) | *Orientia tsutsugamushi* (2.9%) |
|  | Herpes simplex virus-2 (4.6%) | *S. pneumoniae* (5.8%) | Dengue virus (2.5%) |
|  | *S. pneumoniae* (3.4%) | Enterovirus (4.5%) | *Leptospira* (2.3%) |
| **Case fatality rate in adults** | 5.6% | 11.8% | 28.4% |
| **Case fatality rate in children** | 2.7% | 6.7% | 22.5% |

**References**

1. National Healthcare Safety Network. CDC/NHSN surveillance definitions for specific types of infections. Centers for Disease Control and Prevention. Published January 2021. Updated January 2021. Accessed September 18, 2021. <https://www.cdc.gov/nhsn/pdfs/pscmanual/17pscnosinfdef_current.pdf>. .

2. Britton PN, Eastwood K, Paterson B, Durrheim DN, Dale RC, Cheng AC, et al. Consensus guidelines for the investigation and management of encephalitis in adults and children in Australia and New Zealand. Intern Med J. 2015;45(5):563-76. Epub 2015/05/09. doi: 10.1111/imj.12749. PubMed PMID: 25955462.

3. Graus F, Titulaer MJ, Balu R, Benseler S, Bien CG, Cellucci T, et al. A clinical approach to diagnosis of autoimmune encephalitis. Lancet Neurol. 2016;15(4):391-404. Epub 2016/02/20. doi: 10.1016/S1474-4422(15)00401-9. PubMed PMID: 26906964.

4. Ho Dang Trung N, Le Thi Phuong T, Wolbers M, Nguyen Van Minh H, Nguyen Thanh V, Van MP, et al. Aetiologies of central nervous system infection in Viet Nam: a prospective provincial hospital-based descriptive surveillance study. PLoS One. 2012;7(5):e37825. Epub 2012/06/05. doi: 10.1371/journal.pone.0037825. PubMed PMID: 22662232; PubMed Central PMCID: PMCPMC3360608.

5. Dubot-Pérès A, Mayxay M, Phetsouvanh R, Lee SJ, Rattanavong S, Vongsouvath M, et al. Management of Central Nervous System Infections, Vientiane, Laos, 2003-2011. Emerg Infect Dis. 2019;25(5):898-910. doi: 10.3201/eid2505.180914. PubMed PMID: 31002063.
