# Supplementary material for: The epidemiology and outcomes of central nervous system infections in Far North Queensland, tropical Australia; 2000-2019
Source: PLoS One. 2022 Mar 21;17(3):e0265410. doi: 10.1371/journal.pone.0265410 (PMC8936475; doi:10.1371/journal.pone.0265410)
Supplement: S1 File — (DOCX) [file pone.0265410.s013.docx]

**S1 File. Case definitions of CNS infections.**

Diagnosis of meningitis requires at least one of the following criteria:

- Organism identified from cerebrospinal fluid (CSF)
- The presence of two of the following: fever or headache, meningeal signs or cranial nerve signs (and hypothermia, apnoea, bradycardia or irritability for those under <1 year of age)

And one of the following: increased white blood cell (WBC) count, increased protein and decreased glucose in CSF, an organism on Gram stain or culture, or diagnostic serology [1].

Diagnosis of encephalitis requires:

- Presence of decreased or altered level of consciousness, lethargy or personality change lasting at least 24 hours and exclusion of encephalopathy due to other non-infectious aetiologies
- Two of the following for possible encephalitis, three of the following for probable or confirmed encephalitis: fever, seizures, new onset focal neurological findings, CSF WBC count >10 x 10^6^/L, abnormal neuroimaging and electroencephalography [2].

Diagnosis of brain abscess requires at least one of the following criteria:

- Organism identified from brain tissue
- Abscess on gross anatomic or histopathologic exam
- Two of the following: headache, dizziness, fever, localizing neurologic signs, changing level of consciousness or confusion.

And one of the following: organism on microscopic examination of brain or abscess tissue, neuroimaging evidence of infection, or diagnostic serology [1].

Diagnosis of spinal abscess or infection requires at least one of the following criteria:

- Organism identified from abscess
- Abscess or other evidence of spinal infection on anatomic or histopathologic exam
- One of the following: fever, back pain or tenderness, radiculitis, paraparesis or paraplegia

And imaging evidence of spinal abscess/infection [1].
