# Supplementary material for: The epidemiology and outcomes of central nervous system infections in Far North Queensland, tropical Australia; 2000-2019
Source: PLoS One. 2022 Mar 21;17(3):e0265410. doi: 10.1371/journal.pone.0265410 (PMC8936475; doi:10.1371/journal.pone.0265410)
Supplement: S2 File — (DOCX) [file pone.0265410.s014.docx]

**S2 File. Case definitions of autoimmune encephalitis.**

Diagnosis of possible autoimmune encephalitis requires all three of the following criteria:

1. Subacute onset (rapid progression of less than 3 months) of working memory deficits (short-term memory loss), altered mental status, or psychiatric symptoms
2. At least one of the following:

- New focal CNS findings
- Seizures not explained by a previously known seizure disorder
- CSF pleocytosis (white blood cell count of more than five cells per mm^3^)
- MRI features suggestive of encephalitis

1. Reasonable exclusion of alternative causes^a^ [3]

Diagnosis of definite autoimmune limbic encephalitis requires all four of the following criteria:

1. Subacute onset (rapid progression of less than 3 months) of working memory deficits, seizures, or psychiatric symptoms suggesting involvement of the limbic system
2. Bilateral brain abnormalities on T2-weighted fluid-attenuated inversion recovery MRI highly restricted to the medial temporal lobes
3. At least one of the following:

- CSF pleocytosis (white blood cell count of more than five cells per mm^3^)
- EEG with epileptic or slow-wave activity involving the temporal lobes

1. Reasonable exclusion of alternative causes^a^ [3]

Diagnosis of definite acute disseminated encephalomyelitis requires all five of the following criteria:

1. A first multifocal, clinical CNS event of presumed inflammatory demyelinating cause
2. Encephalopathy that cannot be explained by fever
3. Abnormal brain MRI:

- Diffuse, poorly demarcated, large (>1–2 cm) lesions predominantly involving the cerebral white matter
- T1-hypointense lesions in the white matter in rare cases
- Deep grey matter abnormalities (eg, thalamus or basal ganglia) can be present

1. No new clinical or MRI findings after 3 months of symptom onset
2. Reasonable exclusion of alternative causes^a^ [3]

Diagnosis of probable anti-NMDA receptor encephalitis requires all three of the following criteria:

1. Rapid onset (less than 3 months) of at least four of the six following major groups of symptoms:

- Abnormal (psychiatric) behaviour or cognitive dysfunction
- Speech dysfunction (pressured speech, verbal reduction, mutism)
- Seizures
- Movement disorder, dyskinesias, or rigidity/abnormal postures
- Decreased level of consciousness
- Autonomic dysfunction or central hypoventilation

1. At least one of the following laboratory study results:

- Abnormal EEG (focal or diffuse slow or disorganised activity, epileptic activity, or extreme delta brush)
- CSF with pleocytosis or oligoclonal bands

1. Reasonable exclusion of other disorders^a^

Diagnosis can also be made in the presence of three of the above groups of symptoms accompanied by a systemic teratoma

Diagnosis of definite anti-NMDA receptor encephalitis requires the presence of one or more of the six major groups of symptoms and IgG anti-GluN1 antibodies, after reasonable exclusion of other disorders^a^ [3]

Diagnosis of probable Bickerstaff’s brainstem encephalitis requires both of the following criteria:

1. Subacute onset (rapid progression of less than 4 weeks) of all the following symptoms:

- Decreased level of consciousness
- Bilateral external ophthalmoplegia
- Ataxia

1. Reasonable exclusion of alternative causes^a^

Diagnosis of definite Bickerstaff’s brainstem encephalitis requires the presence of positive IgG anti-GQ1b antibodies even if bilateral external ophthalmoplegia is not complete or ataxia cannot be assessed, or if recovery has occurred within 12 weeks after onset. [3]

Diagnosis of autoantibody-negative but probable autoimmune encephalitis requires all four of the following criteria:

1. Rapid progression (less than 3 months) of working memory deficits (short-term memory loss), altered mental status, or psychiatric symptoms
2. Exclusion of well defined syndromes of autoimmune encephalitis (eg, typical limbic encephalitis, Bickerstaff’s brainstem encephalitis, acute disseminated encephalomyelitis)
3. Absence of well characterised autoantibodies in serum and CSF, and at least two of the following criteria:

- MRI abnormalities suggestive of autoimmune encephalitis[^*^](https://www.ncbi.nlm.nih.gov/pmc/articles/PMC5066574/#FN9)
- CSF pleocytosis, CSF-specific oligoclonal bands or elevated CSF IgG index, or both[^*^](https://www.ncbi.nlm.nih.gov/pmc/articles/PMC5066574/#FN9)
- Brain biopsy showing inflammatory infiltrates and excluding other disorders (eg, tumour)

1. Reasonable exclusion of alternative causes^a^ [3]

^a^CNS infections, septic encephalopathy, metabolic encephalopathy, drug toxicity, cerebrovascular disease, neoplastic disorders, Creutzefeldt-Jakob disease, epileptic disorders, rheumatologic disorders, Kleine-Levin, Reye syndrome, mitochondrial diseases, inborn errors of metabolism
