# Supplementary material for: The epidemiology and outcomes of central nervous system infections in Far North Queensland, tropical Australia; 2000-2019
Source: PLoS One. 2022 Mar 21;17(3):e0265410. doi: 10.1371/journal.pone.0265410 (PMC8936475; doi:10.1371/journal.pone.0265410)
Supplement: Attachment — Submitted filename: PONE21-35660.pdf [file pone.0265410.s016.pdf]

The authors try to highlight the epidemiological profiles and clinical outcomes of patients who were diagnosed with central nervous system infections in Far North Queensland, tropical Australia. The data were retrospectively collected from 1st January 2000 until 31st December 2019. I find the manuscript is too verbose and lack of novelty. The abstract was not comprehensively written. For example, the aim of the study was not properly written in the background with lack of problem statement. Methodology was not very clear in terms of the lab tests/methods used for the identification of the pathogens.

Several variables should be defined as follows: tropical pathogens, indigenous communities, not identified pathogens (Table 1), Indigenous Australians, Local Indigenous, disability outcomes, and residual deficits.

Why the first letter of “Indigenous” is capitalized, especially when it is written as Indigenous communities or Indigenous status.

Methods:

The inclusion and exclusion criteria were not clearly mentioned. Were repeat samples included? Were patients with coinfections included?

Line 142-143: “The patients’ demographics, clinical features, comorbidities, and outcomes were collected from the medical records of cases between January 1, 2015, and December 31, 2019” There is a big gap on the data collected between 2000 to 2014, which may affect the statistical analysis in general.

Line 151 -153: the statement should be cited.

Results:

The analysis was affected by different denominators used and this has created a lot of confusion to the readers.

Table 2: what is the purpose of comparing indigenous versus non-indigenous? This was not highlighted in the introduction.

Species should not be italicised.

About enterovirus, was it only one genus identified? What method was used for the identification?

There were too many tables provided, which I think some are not necessary especially the impact of residential address. It would be good if geographic information system is utilized.

Line 312-313: statistical results should be provided.

Discussion:

Line 379: Spelling error was observed, N. meningitides

Table 9 should be de-tabulated, and the authors should use all the points for the discussion. The table is more appropriate for a review paper.

Epidemiological parameters were not critically discussed in relation to CNS infections. Lack of references for the comparison was observed.
