# Supplementary material for: The epidemiology and outcomes of central nervous system infections in Far North Queensland, tropical Australia; 2000-2019
Source: PLoS One. 2022 Mar 21;17(3):e0265410. doi: 10.1371/journal.pone.0265410 (PMC8936475; doi:10.1371/journal.pone.0265410)
Supplement: Attachment — Submitted filename: Response to Reviewers.docx [file pone.0265410.s018.docx]

**Response to Reviewers**

We thank the editorial staff and reviewers for the time that they have taken to review our manuscript and the helpful comments that they have made to improve the work. Please find below our point-by-point responses to their comments. For clarity, the editorial staff and reviewers’ comments are presented in blue normal text, while our responses are in black normal text. Any modified – or highlighted – text is presented as black and italicized.

REVIEWERS COMMENTS

Reviewer #2: The authors have adequately addressed all the previous comments by the reviewer. The revised manuscript has some merits for the reader as the results are well presented and well-concluded. Congratulations!

Response: Thank you very much.

Reviewer #3: An important retrospective study is presented. The authors have responded well to the comments from the reviewers.
The manuscript is now acceptable for publication. I have only a few minor comments and recommendations:
Recommend change of the end of the title from " a 20 -years retrospective study" to " 2000- 2019"
In the Results section in Abstract: Remove all the quata of 725 from 561/725 etc and present only percentages. The number 725 is presented in the beginning of the results.
Remove decimals on percentages when n<100 :
Rows 268, 271, 280, 290, 292, 293, 308, 319, 321, 336 and on
Table 4 on the group Remote areas (n=80)
Table 8 on Died (n= 32)

Response: We thank the reviewer for their comments. This is really a question of style, but we have revised the text as suggested (lines 4, 36-58, 251, 256-258, Table 4, 294, 297, 306, 316, 321, 323, 324, 331, 343, 354, 356, 363, 376, Table 8).
